# Higher magnesium levels are associated with better glycaemic control and diabetes remission post-bariatric surgery

**DOI:** 10.1186/s12902-022-01210-4

**Published:** 2022-12-06

**Authors:** Silva MM, Neves JS, Borges-Canha M, Mendes AP, Fonseca MJ, Mendonça F, Ferreira MJ, Salazar D, Pedro J, Guerreiro V, Lau E, Varela A, Freitas P, Carvalho D, CRIO group

**Affiliations:** 1 Serviço de Endocrinologia, Diabetes E Metabolismo, Centro Hospitalar Universitário de São João, Porto, Portugal; 2 Faculty of Medicine, University of Porto, Porto, Portugal; 3 Investigação E Inovação Em Saúde (i3s), Faculdade de Medicina da Universidade Do Porto, Porto, Portugal; 4 Departamento de Cirurgia E Fisiologia, Faculdade de Medicina da Universidade Do Porto, Alameda Prof. Hernâni Monteiro, 4200-319 Porto, Portugal; 5 Medical and Performance Department, Sporting Clube de Portugal, Estrada da Malhada de Meias, Barroca d’Alva, 2890-529 Lisboa, Portugal; 6 Institute of Public Health of the University of Porto (ISPUP), Porto, Portugal

**Keywords:** Magnesium, Bariatric surgery, Obesity, Type 2 diabetes *mellitus*

## Abstract

**Background:**

Low Magnesium (Mg) dietary intake has been associated with increased risk of type 2 diabetes *mellitus* (T2DM). Furthermore, in patients with T2DM, hypomagnesemia is associated with worst glycaemic control. Bariatric surgery (BS) remains the most effective treatment in severe obesity and also provides resolution/improvement of T2DM. Our aim is to evaluate the association between Mg supplementation post-BS and Mg serum levels with diabetes status after BS.

**Methods:**

We performed an observational study on patients with obesity and T2DM who underwent BS. Data was assessed pre-BS and one-year post-BS.

**Results:**

We included a total of 403 patients with T2DM. At baseline, 43.4% of the patients had Mg deficiency. Pre-BS, patients with Mg deficiency had poorer glycaemic control – HbA1c 7.2 ± 1.6% *vs* 6.4 ± 1.0% (*p* < 0.001), fasting plasma glucose 146.2 ± 58.8 mg/dL *vs* 117.5 ± 36.6 mg/dL (*p* < 0.001) and were under a greater number of anti-diabetic drugs 1.0 (IQR 0–2.0) *vs* 1.0 (IQR 0–1.0) (*p *= 0.002). These findings persisted at one-year post-BS. At the first-year post-BS, 58.4% of the patients had total remission of T2DM and 4.1% had partial remission. Patients without Mg deficiency at one-year post-BS had higher rates of total and partial remission. Higher serum Mg levels at baseline is an independent predictor of total T2DM remission (*p* < 0.0001). The optimal cut-off of baseline Mg to predict total T2DM remission was 1.50 mg/dL with a sensitivity of 73% and a specificity of 58% (area under ROC = 0.65). Patients that were under Mg supplementation post-BS had serum Mg values, glycaemic control and total remission of T2DM similar to patients non-supplemented.

**Conclusion:**

In patients with T2DM submitted to BS, higher Mg serum levels at baseline and 1-year after BS were associated with better glycaemic control and higher rates of total T2DM remission at the first year post-BS.

**Supplementary Information:**

The online version contains supplementary material available at 10.1186/s12902-022-01210-4.

## Background

Magnesium (Mg) acts as a cofactor of enzymes involved in glucose metabolism, protein production and nucleic acid synthesis [1, 2]. Mg balance is regulated by an interaction between intake, intestinal absorption, renal excretion and exchange from bone (not completely available in cases of Mg deprivation) [2]. Because of the daily loss of Mg in faeces, urine and sweat, humans require a continuous supply from exogenous sources by dietary intake – recommended dietary allowance of 420 mg/day for adult men and 320 mg/day for adult women [3, 4]. Mg intake needs are higher in certain periods of life, varying across age and sex.

Mg deficiency is relatively common in general population [5, 6] and the primary cause of hypomagnesemia is often insufficient dietary intake. In Europe and in the United States, the daily allowance of Mg is not accomplished in a large proportion of people [7].

Dietary Mg is mostly absorbed by the duodenum and jejunum via passive paracellular transport. The intestinal absorption is not directly proportional to Mg intake, but depends on body’s Mg status [8]. Serum or plasma Mg concentration is the most common used biomarker to assess Mg metabolism abnormalities in clinical practice [9]. The normal range of serum Mg is 0.76–1.15 mmol/L, approximately 1.52–2.30 mEq/L [2]. Although hypomagnesemia is not always present in cases of Mg deficiency, it is usually indicative of an important systemic Mg deficiency [10].

Literature suggests an association between Mg and cardiovascular risk. Mg dietary intake is inversely associated with the incidence of several cardio-metabolic conditions, namely type 2 diabetes *mellitus* (T2DM) [2, 4, 11–19]. This finding suggests that increased consumption of Mg-rich foods or Mg supplements may reduce the risk of T2DM [11, 14–17]. Some authors suggest that Mg supplementation could improve glycaemic control in T2DM patients [20–22], while, at the same time, other studies show no significant effects of Mg supplementation on T2DM [23, 24]. On the other hand, the relation between Mg serum levels and T2DM is more intriguing [10, 25]. T2DM is often accompanied by hypomagnesemia, especially in older patients, with poorly controlled glycaemic profiles, with longer duration of the disease or presence of micro or macrovascular chronic complication [26–30]. The incidence of hypomagnesemia in patients with T2DM ranges from 13.5 to 47.7% compared with 2.5 to 15% in healthy control subjects [23, 31, 32]. In a 2017 study, authors observed a prevalence of 30.6% in T2DM patients [33].

T2DM and obesity are increasingly common and major global health problems [34]. Adults with obesity are at increased risk for developing major diseases, such as T2DM [35, 36]. Bariatric surgery (BS) remains the most effective treatment in severe obesity [37] and also provides T2DM remission or improvement of glucometabolic status (which became a recommendation to BS in individuals unable to achieve adequate glycaemic control with oral or injectable medications) [38]. After Roux-en-Y Gastric Bypass (RYGB), remission rates between 43.2% and 84% have been reported, with heterogeneity regarding the definition of diabetes remission [39]. Several studies have tried to identify predictive factors for T2DM remission after BS. Due to the previously described relationship between Mg and T2DM, the post-bariatric assessment of Mg status has been evaluated recently [40], and patients with T2DM that achieved remission after RYGB had higher Mg serum values when compared to patients that didn’t achieve remission.

After BS, micronutrient deficiencies are one of the most common and compelling problems and supplementation is recommended [29]. On the other hand, the majority of studies show a decrease in hypomagnesemia in post-bariatric patients [41–47].

As mentioned previously, in general, higher Mg intake is associated with lower risk of T2DM and better glycaemic control and, on the other hand, hypomagnesemia occurs, typically, in patients with poor glycaemic control. Taking into account that numerous micronutrient deficiencies are more common after BS, the aim of this study is to evaluate the association between Mg supplementation post-BS and Mg serum levels with T2DM glucometabolic status and remission. To the best of our knowledge, this is the first study aiming to assess the relationship between Mg supplementation post-BS and T2DM glycaemic control and remission in post-bariatric patients.

## Methods

This study was approved by Ethical Committee for Health of Centro Hospitalar Universitário de São João, Porto, Portugal. For this type of study formal consent is not required in accordance with the national legislation and the institutional requirements [48]. All procedures performed in this study involving human participants were in accordance with the ethical standards of the institutional and/or national research committee and with the 1964 Helsinki declaration and its later amendments or comparable ethical standards.

### Study design and included population

This is a retrospective observational study according to Strengthening the Reporting of Observational Studies in Epidemiology (STROBE) Statement [49]. We included a population of patients with obesity (body mass index ≥ 35 kg/m2 plus comorbidities related to obesity, and body mass index ≥ 40 kg/m2) submitted to BS (Roux-en-Y gastric bypass or sleeve gastrectomy) and followed between January 2010 and June 2017. We included all patients with T2DM criteria pre-operatively and available serum Mg values submitted to BS during this period. We excluded patients without available glycated haemoglobin (HbA1c) or fasting plasma glucose (FPG) in pre or post-BS evaluations. All study participants were treated according to the usual clinical care.

### Clinical and biochemical parameters evaluated

**Fig. 1:**
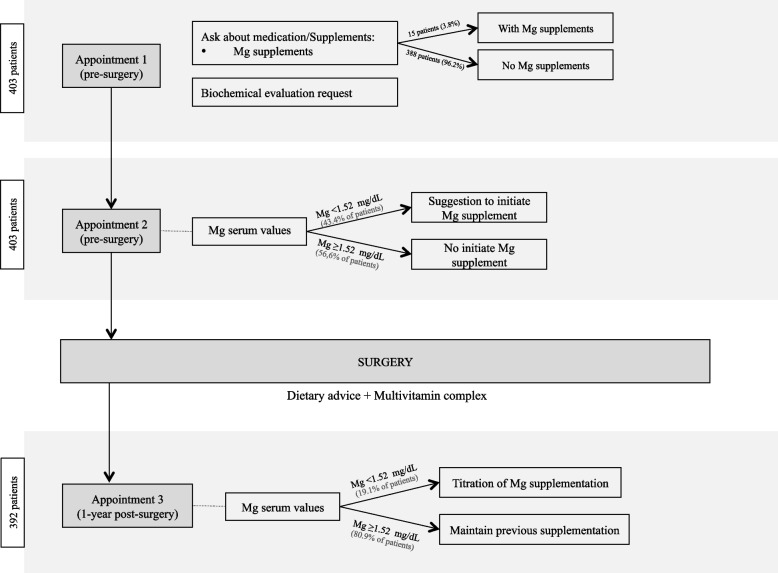
Magnesium supplementation and serum evaluation during pre and post-surgery evaluations

Patients were evaluated pre-operatively and 1-year post-BS. The following pre and post-BS parameters were recorded: age, sex, body mass index (BMI), serum Mg, Mg supplementation, FPG, HbA1c, plasma glucose after a 75-g oral glucose tolerance test, use of anti-diabetic drug and fasting C-peptide. We defined T2DM based in part in criteria from the American Diabetes Association: FPG ≥ 126 mg/ dL, HbA1c ≥ 6.5%, 2-h plasma glucose after a 75-g oral glucose tolerance test ≥ 200 mg/dL, or the use of anti-diabetic medications [50]. Complete T2DM remission was defined as HbA1C < 6.0% and no anti-diabetic medication use and partial T2DM remission was defined as HbA1C < 6.5% and no anti-diabetic medication use (35). Normal serum Mg was defined as serum Mg ≥ 1.52 mEq/L [2]. Mg was measured on serum, obtained from blood samples during clinical evaluations. All patients were evaluated pre-surgery and questioned about Mg supplementation (Fig. 1). Pre-surgery supplementation data refer to this appointment (Appointment 1). After biochemical evaluation, if hypomagnesemia was present, patients were instructed to start on Mg supplementation (Appointment 2). The compliance to this supplementation was not evaluated. After surgery, all patients were given the same kind of dietary advice and were also recommended to take a multivitamin complex (containing, at least, 100 mg Mg oxide). At 1-year post-BS appointment (Appointment 3), compliance to post-BS supplementation was evaluated. If patients maintained hypomagnesemia, adjustements for Mg supplementation were made. The supplemented group was doing between 100–450 mg of elemental Mg daily. Dietary Mg content was not evaluated. Data referring Mg supplementation post-BS referred to supplementation compliance between surgery and this appointment. Regarding BS, patients were submitted to RYGB or SG. RYGB was performed as a standardized technique, with a small gastric pouch (30 cc), calibrated 36 Fr gastro-jejunal anastomosis, biliopancreatic limb of 100 cm, and alimentary limb of 120 cm.

### Statistical analysis

Comparisons between groups were made using *t tests* and the ANOVA test. We performed the analysis unadjusted and adjusted for sex, age, BMI and estimated glomerular filtration rate (eGFR) in pre-surgery evaluation and adjusted for sex, age, BMI, eGFR, weight variation and type of surgery in post-BS evaluation. A multivariable logistic regression model was performed to evaluate predictors of T2DM remission. We defined the optimal cut-off of baseline Mg to predict complete T2DM remission using a univariate receptor operating characteristic (ROC) curve and Youden index. Two-sided *p* < 0.05 was considered statistically significant. Statistical analysis was performed with Stata software, Version 14.1 (StataCorp).

## Results

### Baseline population characteristics

In Table 1 we show the clinical and demographic characteristics of the population included at baseline. We included 403 individuals, from which 79.4% (*n* = 320) were females. The individuals were in average 47.0 ± 10.1 years old and their average BMI was 45.0 ± 6.7 kg/m2. 68,7% (*n* = 277) of patients were submitted to RYGB and 31.3% (*n* = 126) were submitted to sleeve gastrectomy (SG). Ninety-six percent of the included individuals were not on oral Mg supplementation, and the mean serum Mg was 1.53 ± 0.17 mEq/L. Across follow-up, patients in the supplemented group were doing between 100–450 mg of elemental Mg daily, either by multivitamin complex, Mg salt or both.

**Table 1:** Clinical and biochemical characteristics of the enrolled patients before surgery

	Patients before surgery				
	All	Mg < 1.52 mEq/L	Mg ≥ 1.52 mEq/L	*p* value	*p* value (adjusted)
	*n* = 403	(*n* = 175)	(*n* = 228)		
Sex, n (%)				0.32	
Female	320 (79.4%)	143 (81.7%)	177 (77.6%)		
Male	83 (20.6%)	32 (18.3%)	51 (22.4%)		
Age (years), mean (SD)	47.0 (10.1)	47.6 (10.0)	46.5 (10.2)	0.28	
Type of surgery, n (%)				0.48	
Roux-en-Y gastric bypass	277 (68.7%)	117 (66.9%)	160 (70.2%)		
Sleeve gastrectomy	126 (31.3%)	58 (33.1%)	68 (29.8%)		
Body mass index (kg/m2), mean (SD)	45.0 (6.7)	45.2 (6.6)	44.9 (6.8)	0.71	
Serum Mg (mEq/L), mean (SD)	1.53 (0.17)	1.39 (0.12)	1.64 (0.1)	** < 0.001**	** < 0.001**
Serum Mg < 1.52 mEq/L, n (%)	175 (43.4%)				
Vitamin D (ng/mL), mean (SD)	15.6 (8.5)	17.7 (9.4)	14.2 (8.0)	**0.03**	0.77
HbA1c (%), mean (SD)	6.8 (1.4)	7.2 (1.6)	6.4 (1.0)	** < 0.001**	** < 0.001**
Fasting plasma glucose (mg/dL), mean (SD)	130.1 (49.6)	146.2 (58.8)	117.5 (36.3)	** < 0.001**	** < 0.001**
Fasting plasma insulin (mU/L), mean (SD)	25.2 (20.1)	24.8 (21.4)	25.5 (19.3)	0.81	0.47
Fasting plasma C-Peptide (ng/mL), mean (SD)	4.4 (1.7)	4.4 (1.9)	4.5 (1.5)	0.85	0.90
HOMA-IR, mean (SD)	8.2 (7.6)	9.0 (9.2)	7.6 (6.3)	0.25	0.92
No. of anti-diabetics, median (IQR)	1.0 (0–1.0)	1.0 (0–2.0)	1.0 (0–1.0)	**0.003**	**0.002**
Oral magnesium supplementation, n (%)				**0.003**	**0.001**
Yes	15 (3.8%)	12 (7.1%)	3 (1.3%)		
No	388 (96.2%)	163 (92.9%)	225 (98.7%)		
Total cholesterol (mg/dL), mean (SD)	201.2 (43.3)	194.5 (42.3)	206.5 (43.5)	**0.006**	**0.003**
LDL-cholesterol (mg/dL), mean (SD)	124 (37.6)	116.5 (36.5)	129.9 (37.5)	** < 0.001**	** < 0.001**
HDL-cholesterol (mg/dL), mean (SD)	48.1 (10.8)	48.0 (11.6)	48.1 (10.1)	0.96	0.75
Triglycerides (mg/dL), mean (SD)	166.6 (99.9)	179.9 (117.5)	156.3 (82.5)	**0.019**	**0.011**

*Mg* Magnesium, *HbA1c* Glycated haemoglobin

### Mg serum values and metabolic control at baseline and 1 year after surgery

In Table 1 and 2 we show the metabolic control at baseline and 1 year after surgery according to Mg serum levels. At baseline, 43.4% of the patients had Mg deficiency. These patients had worst glycaemic control, namely: higher HbA1c 7.2 ± 1.6% *vs* 6.4 ± 1.0% (*p* < 0.001), higher FPG 146.2 ± 58.8 mg/dL *vs* 117.5 ± 36.6 mg/dL (*p* < 0.001) and were under a greater number of anti-diabetic drugs 1.0 [IQR 0–2.0] *vs* 1.0 [IQR 0–1.0] (*p* = 0.002). Although patients with hypomagnesemia had higher insulin resistance (HOMA-IR 9.0 ± 9.2 *vs* 7.6 ± 6.3) this had not statistical significance (*p* = 0.92).

**Table 2:** Magnesium status and metabolic control 1-year post-surgery

	1-year post-surgery			
	Mg < 1.52 mEq/L	Mg ≥ 1.52 mEq/L	*p* value	*p* value (adjusted)
	(*n* = 75)	(*n* = 317)		
Vitamin D (ng/mL), mean (SD)	25.7 (12.1)	24.4 (13.8)	0.50	0.22
HbA1c (%), mean (SD)	5.8 (0.7)	5.5 (0.6)	** < 0.001**	**0.01**
No. of anti-diabetics, median (IQR)	1.0 (0.5–2.0)	1.0 (0–1.0)	**0.03**	0.05
Fasting plasma glucose (mg/dL), mean (SD)	96.8 (25.4)	89.8 (15.8)	**0.003**	**0.02**
Fasting plasma insulin (mU/L), mean (SD)	7.6 (4.5)	7.3 (4.5)	0.54	0.63
Fasting plasma C-Peptide (ng/mL), mean (SD)	2.2 (0.9)	2.2 (0.7)	0.98	0.30
HOMA-IR, mean (SD)	1.8 (1.2)	1.6 (1.1)	0.30	0.48
Total cholesterol (mg/dL), mean (SD)	182.4 (40.5)	180.3 (35.9)	0.67	0.71
LDL-cholesterol (mg/dL), mean (SD)	106.4 (32.6)	106.1 (30.0)	0.94	0.85
HDL-cholesterol (mg/dL), mean (SD)	55.5 (12.0)	55.5 (11.9)	0.99	0.96
Triglycerides (mg/dL), mean (SD)	107.9 (39.7)	97.6 (39.6)	**0.046**	0.28

*Mg* Magnesium, *HbA1c* Glycated haemoglobin

At 1-year post-BS, the prevalence of Mg deficiency was lower when compared with pre-surgical moment (19.1% vs 43.4% of patients) and the mean serum Mg was higher compared to baseline – 1.62 ± 0.14 mEq/L (*n* = 392) *vs* 1.53 ± 0.17 mEq/L (*n* = 403). 11 patients were withdrawn due to missing Mg serum values, HbA1c or FPG. Regarding glycaemic profile, we found results overlapping the baseline findings. Patients with Mg deficiency had higher HbA1c 5.8 ± 0.7% *vs* 5.5 ± 0.6% (*p* = 0.01), higher FPG 96.8 ± 25.4 mg/dL *vs* 89.8 ± 15.8 mg/dL (*p* = 0.02) and took a higher number of anti-diabetic drugs 1.0 [IQR 0.5–2.0] *vs* 1.0 [IQR 0–1.0] (*p* = 0.04). Insulin resistance was not significantly higher in Mg-deficient patients – HOMA-IR 1.8 ± 1.2 *vs* 1.6 ± 1.1 (*p* = 0.48).

### Mg supplementation post-BS and metabolic control at baseline and 1 year after surgery

At the initial assessment, the majority of patients did not take any Mg supplementation (96.2%). After surgery, 78.0% of patients were under Mg supplementation.

In Table 3 we evaluate the differences in metabolic parameters of patients with and without Mg supplementation post-BS. In adjusted analysis, no differences were registered between groups.

**Table 3:** Metabolic profile and type 2 diabetes *mellitus* remission according to oral magnesium supplementation

	1 year after surgery			
	Oral Mg supplementation	Without any supplementation	*p* value	*p* value (adjusted)
**Variables (*n* = 454)**				
No. of patients (%)	354 (78.0%)	100 (22.0%)		
Serum magnesium (mEq/L), mean (SD)	1.63 (0.14)	1.62 (0.17)	0.53	0.84
Vitamin D (ng/mL), mean (SD)	25.0 (10.5)	24.6 (21.2)	0.81	0.88
Fasting plasma glucose (mg/dL), mean (SD)	89.5 (16.4)	93.3 (18.4)	0.06	0.36
HbA1c (%), mean (SD)	5.5 (0.5)	5.7 (0.9)	**0.007**	0.12
Fasting plasma insulin (mU/L), mean (SD)	7.5 (4.7)	7.2 (4.5)	0.66	0.29
Fasting plasma C-Peptide (ng/mL), mean (SD)	2.3 (0.7)	2.1 (0.8)	0.20	0.15
No. of antidiabetics, median (IQR)	1.0 (0–1.0)	1.0 (0–1.0)	0.69	0.55
HOMA-IR, mean (SD)	1.7 (1.2)	1.7 (1.0)	0.90	0.33
No. Patients with total remission, n (%)	196 (62.0%)	42 (48.3%)	0.06	0.18
No. Patients with partial remission, n (%)	13 (4.1%)	4 (4.6%)		

### T2DM remission

At 1-year post-BS, 58.4% (*n* = 229) of the patients had total remission of T2DM and 4.1% (*n* = 16) had partial remission of T2DM. Concerning serum Mg levels, patients with total remission had higher levels when compared with patients that didn’t remit—1.65 ± 0.13 mEq/L *vs* 1.58 ± 0.16 mEq/L (*p* < 0.001).

In Table 4, we show T2DM remission according to type of surgery with mean serum Mg values for each type of remission. In patients submitted to RYGB (*n* = 273), around 62% had total remission. These patients had higher Mg serum values compared to patients that had no T2DM remission—1.64 ± 0.14 mEq/L *vs* 1.59 ± 0.16 mEq/L (*p* = 0.01). In patients submitted to SG, we had 51.3% of total T2DM remission. Higher serum Mg values were observed in the group of patients achieving T2DM total remission.

**Table 4:** Type 2 diabetes mellitus remission according to type of surgery

	RYGB				SG			
	(*n* = 273)				(*n* = 119)			
	TR	PR	NR	*p* value	TR	PR	NR	*p* value
No. of patients, n (%)	168 (61.5%)	13 (4.8%)	92 (33.7%)		61 (51.3%)	3 (2.5%)	55 (46.2%)	
Serum magnesium (mEq/L), mean (SD)	1.64 (0.14)	1.66 (0.17)	1.59 (0.16)	**0.01**	1.67 (0.11)	1.59 (0.08)	1.56 (0.15)	** < 0.001**

*RYGB* Roux-en-Y gastric bypass, *SG* Sleeve gastrectomy, *TR* Total remission, *PR* Partial remission, *NR* No remission

Patients that were under Mg supplementation post-BS did not have higher rates of total T2DM remission – 62.0% *vs* 48.3% (*p* = 0.18).

In Table 5, we show a multivariate logistic regression model of predictors of T2DM remission 1-year after surgery. Mg serum levels at baseline are an independent predictor of T2DM total remission [OR 1.37 (1.17–1.61), *p* < 0.001]. In our analysis, type of surgery (SG vs RYGB) was not an independent predictor of T2DM remission [OR 0.90 (0.51–1.60), *p* = 0.72].

**Table 5:** Multivariable logistic regression model of predictors of type 2 diabetes mellitus remission 1-year after surgery

**Variable (*n* = 306)**	Odds ratio	*p* value
Serum magnesium at baseline, per 0.1 mEq/L	1.37 (1.17–1.61)	** < 0.0001**
Type of surgery, SG (vs RYGB)	0.90 (0.51–1.60)	0.72
Sex, male	0.77 (0.39–1.52)	0.45
Age, years	0.94 (0.91–0.96)	** < 0.0001**
Pre-surgery BMI, kg/m^2^	0.94 (0.90–0.99)	0.01
Weight variation, kg	1.02 (0.99–1.05)	0.09
eGFR, mL/min/m^2^	1.02 (1.01–1.04)	1.01

*SG* Sleeve gastrectomy, *RYGB* Roux-en-Y Gastric Bypass, *BMI* Body mass index, *eGFR* Estimated glomerular filtration rate

The optimal cut-off of baseline Mg to predict total T2DM remission was 1.50 mg/dL with a sensitivity of 73% and a specificity of 58% (area under ROC = 0.65).

## Discussion

In this study, we report four main findings. First, patients with Mg deficiency (pre and post-BS) had worst glycaemic control in pre and post-BS evaluations. Second, patients with normal serum Mg levels post-BS had higher chances of T2DM total remission on first year post-BS. Third, serum Mg values at baseline were an independent predictor of T2DM remission (cut-off was 1.50 mg/dL). Fourth, patients under Mg supplementation post-BS did not differ from patients without Mg supplementation on glycaemic control, insulin sensitivity and T2DM total remission on first year post-BS.

When we focus on Mg dietary intake (either by food or supplements), many epidemiological studies and, more recently, randomized controlled trials, demonstrated an inverse relation between higher dietary Mg content and T2DM [14–19]. In patients with T2DM, higher Mg intake improves metabolic control, target organ damage and cardiovascular risk factors [20–22]; in patients without T2DM, Mg reduces T2DM risk. These studies were conducted in patients non-submitted to BS. In our study, at first year post-BS, Mg-supplemented patients did not differ from non-supplemented patients in Mg serum values, glycaemic control and insulin sensitivity. The compliance to supplementation was self-reported and the supplemented group was doing Mg either by multivitamin complex or Mg salt, with different doses and formulations. This could be a possible explanation for this analysis did not reach statistical significance. Also, we do not know if Mg supplements are equally effective in patients submitted to BS and non-submitted. We cannot compare our results with other studies because, to the best of our knowledge, this is the first study to address this question in bariatric patients.

While some previous studies have reported higher prevalence of Mg deficiency after BS [46], an improvement in Mg status post-BS has been recently demonstrated in patients with and without diabetes [41–44]. Our results corroborated this, with lower hypomagnesemia prevalence and higher mean serum Mg levels in 1-year post-BS, when compared to baseline.

As we stated previously, in patients with diabetes poorly controlled, hypomagnesemia is relatively common [26–30]. In our study, we showed that lower serum Mg levels, pre and post-BS, were associated with worst glycaemic control. The relationship between low serum Mg levels in patients with T2DM might be explained by reduced Mg intake and/or augmented Mg urinary loss [10, 33]. In post-bariatric patients, poor eating habits and intestinal malabsorption are other possibly explanations.

Regarding T2DM remission rates, patients with normal serum Mg values post-BS had higher T2DM total and partial remission rates. When remission rates were evaluated according to the type of surgery, patients with total remission had higher Mg serum levels independently of being submitted to RYGB or SG. In agreement with our results, a recent long-term cohort study [40] showed that serum Mg values increased after RYGB, with higher serum values in patients with T2DM remission, compared with patients that did not achieve remission. These authors suggested that the preoperative circulating Mg status might serve as a predictor for T2DM remission after RYGB. In our study, to study the predictors of T2DM remission at 1-year post-BS, Mg serum levels at baseline were an independent predictor of T2DM total remission (cut-off was 1.50 mg/dL). Type of surgery (SG *vs* RYGB) was not an independent predictor of remission.

There are limitations that should be addressed in our study. As an observational study, the possibility of confounding factors (diet, lifestyle and socioeconomic factors) cannot be excluded. As we stated previously, a possible explanation to Mg supplementation analysis did not reach statistical significance was that patients in the supplemented group were doing Mg supplementation either by multivitamin complex or Mg salt, with different doses (dose of elemental Mg could range between 100 to 450 mg daily), formulations and, possibly, compliance. Also, Mg food-derived intake could not be assessed. Higher consumption of Mg-rich foods has demonstrated to reduce T2DM risk and improve glycaemic control and blood lipids in patients with T2DM [10]. Additionally, we cannot exclude the possibility that other nutrients and/or dietary components correlated with Mg may have been responsible, either partially or entirely, for the observed associations.

To the best of our knowledge, this is the first study aiming to assess the relationship between Mg supplementation/Mg serum levels with glycaemic control and T2DM remission rates after BS. Furthermore, establishing a cut-off point for baseline serum Mg level to predict T2DM remission and the elevated number of patients are additional strengths of our study.

In the future, there is a need to carry out prospective studies to analyze the effect of Mg supplementation in post-bariatric patients with T2DM.

## Conclusion

In patients with T2DM submitted to BS higher Mg serum levels at baseline and 1-year after surgery were associated with better glycaemic control and higher rates of total T2DM remission at the first year post-BS. The optimal cut-off of baseline Mg to predict total T2DM remission was 1.50 mg/dL.

## Supplementary Information

**Additional file 1.** Supplementary information.

## Data Availability

The data is available from the corresponding author upon reasonable request and after approval from the Centro Hospitalar Universitário de São João’s Ethical Committee. This is not a public database and it can be made available only according to an authorization.
