# Supplementary material for: Higher magnesium levels are associated with better glycaemic control and diabetes remission post-bariatric surgery
Source: BMC Endocr Disord. 2022 Dec 6;22:303. doi: 10.1186/s12902-022-01210-4 (PMC9724332; doi:10.1186/s12902-022-01210-4)
Supplement: Supplementary file 1 — Additional file 1. Supplementary information. [file 12902_2022_1210_MOESM1_ESM.pdf]

Supplementary information

CRIO group

All members:

Costa EL[g], Preto J[g], Silva H[g], Pinho A[g], Resende F[g], Freitas P[a,b,c] , Varela A[a,b,c], Souto S[a,b,c], Pedro J[a,b,c], Rodrigues P[a,b,c], Pinto E[h], Macedo M[h], Timóteo S[i], Nunes P[i], Barranha R[i], Magalhães AM[j], Santos A[j], Vieira F[j], Botelho M[j], Santos F[j], Teixeira C[k], Correia F[k], Gil C[k], Melim D[k], Rola M[k]

[a]Serviço de Endocrinologia, Diabetes e Metabolismo, Centro Hospitalar Universitário de São João, Porto, Portugal
[b]Faculty of Medicine, University of Porto, Porto, Portugal
[c]Investigação e Inovação em Saúde (i3s), Faculdade de Medicina da Universidade do Porto, Porto, Portugal
[g]Serviço de Cirurgia Geral, Centro Hospitalar Universitário de São João, Porto, Portugal
[h]Serviço de Gastrenterologia, Centro Hospitalar Universitário de São João, Porto, Portugal
[i]Serviço de Psiquiatria, Centro Hospitalar Universitário de São João, Porto, Portugal
[j]Serviço de Anestesiologia, Centro Hospitalar Universitário de São João, Porto, Portugal
[k]Serviço de Nutrição, Centro Hospitalar Universitário de São João, Porto, Portugal
